# Embryo Implantation: New Molecular Insights in Endometrial Receptivity, Trophoblast Invasion and Signalling—An Introduction

**DOI:** 10.3390/biom16060921

**Published:** 2026-06-22

**Authors:** Hans-Werner Denker, Evdokia Dimitriadis, Lois A. Salamonsen

**Affiliations:** 1 Institut für Anatomie, Universität Duisburg-Essen, 45147 Essen, Germany; 2 Department of Obstetrics, Gynaecology and Newborn Health, University of Melbourne, Melbourne, VIC 3010, Australia; evdokia.dimitriadis@unimelb.edu.au; 3 Centre for Reproductive Health, Hudson Institute of Medical Research, Department of Obstetrics and Gynaecology, Monash University, Melbourne, VIC 3168, Australia; lois.salamonsen@hudson.org.au

Embryo implantation within the uterus is critical for the establishment of pregnancy and is an important issue in infertility. Given the impressive amount of molecular and cellular data that has been accumulated in recent years, it seemed timely to review these findings in a new attempt to form a better understanding of this fascinating biological process. Importantly, our understanding over the past decades has been built upon the development of new techniques, starting with assays for the steroid and protein hormones that drive the endocrine regulation of reproductive organs, through cell biological approaches as cell culture techniques developed, and leading to concepts such as epithelial–mesenchymal transitions (EMT) and stromal cell decidualization. More recently, molecular approaches, such as ‘omics’, single-cell technologies, and sophisticated 3D in vitro models, have enabled a new understanding of the cellular mechanisms and signalling processes involved in implantation. The articles bound together in this Special Issue of *Biomolecules* provide an overview of the present state of knowledge.

Many of the contributions to this volume deal in detail with developing in vitro systems and the progress made in the attempts at modelling the partner components of implantation, the endometrium (and its receptivity) and trophoblast, with emphasis on human model systems. The present issue focuses on the insights gained by applying cell biological concepts, while other areas of actual research, like immune cell adaptation, EV signalling and non-coding RNA regulation, are beyond its scope.

The starting perspective paper by **Hans-Werner Denker (Contribution 1)** critically examines the history of how the dominating cell biological concepts, particularly EMT theory, developed, how they have led us to the current state of the research, in what sense they can or cannot be expected to serve the ongoing research, and to what extent it is justified to modify these concepts. Importantly, comparison with other fields of cell biology, such as tumour cell invasion and the epithelial fusion processes of developmental biology, may prove productive in advancing our understanding of implantation. Regarding endometrial receptivity but also trophoblast invasiveness, it could be useful to reconsider the original concept of partial EMT in the search for the specific programme changes involved in uterine epithelial receptivity that may provide a rational basis for improving therapeutic approaches.

Further focus on the human endometrial epithelium and on changes in polarity, adhesion, cytoskeletal organisation, and the underlying extracellular matrix enabling embryo implantation is provided by **Irmgard Classen-Linke et al. (Contribution 2)**. The adhesion and invasion of the trophoblast via the apical plasma membrane of epithelial cells is addressed as a unique cell biological process coupled with partial EMT. This contribution focuses on the human situation, using cell culture systems to study the interaction between human trophoblast and endometrial cells, emphasising molecular details of cell adhesion and cytoskeletal organisation in the uterine epithelium as important factors of endometrial receptivity. Building on the relatively scarce in vivo data from animal models (e.g., rabbit), more detailed insights into cell biological states, obtained with recently developed in vitro culture systems, are reviewed and discussed.

Over the past decade, the development of robust three-dimensional (3D) in vitro models using human endometrial cells has provided a much deeper understanding than the earlier 2D cell models. **Jenna Douglas and her co-investigators in the Fitzgerald group (Contribution 3)** review recent advances in such systems: endometrial epithelial organoids, trophoblast organoids, and increasingly complex co-culture platforms incorporating stromal, vascular, and trophoblast compartments can effectively model epithelial–stromal crosstalk, decidualisation, angiogenesis, and embryo implantation. Emerging developments include assembloid systems, synthetic and semi-synthetic extracellular matrices, and microfluidic organ-on-a-chip technologies that enable long-term culture, hormonal responsiveness, and patient-specific modelling. These approaches have recapitulated key features of the mid-secretory endometrium, placental villous architecture, trophoblast differentiation, and early implantation events while revealing disease-associated dysfunctions in conditions such as endometriosis, adenomyosis, and polycystic ovarian syndrome. Detailed examination of the existing literature describing the advances in 3D cell culture models of the human endometrium and the early-stage embryo highlights the potential in applying and/or combining many of these approaches to engineer the human endometrial–embryo interface in vitro in the future to study endometrial biology and disease, and their uses for modelling endometrial dysfunction in gynaecological disease, placental function, and implantation.

Ethical and regulatory considerations are critical in any research involving human embryos. **Megan Munsie and Jock Findlay (Contribution 4)** discuss recently developed three-dimensional in vitro models using human material, including human stem cell-based embryo models (hSCBEM) and endometrial models, and how such models could be employed in advancing assisted reproductive technologies and understanding implantation failure. Importantly, the ethical and legal implications are examined, and various governance models are explored that have been proposed to foster responsibility and innovation in this area of research, including, as an example, an Australian regulation that is remarkably different from rulings in most other countries. How and when to involve the public in the development of such technologies and their regulation remain important questions. Similar regulatory issues are raised by **Søren Holm (Contribution 5)** regarding the use of hSCBEM in implantation research. These models are rapidly becoming closer to morphological and functional identity with human embryos. This paper analyses two possible approaches to resolving regulatory issues. The first approach is to maintain consistency with current regulations of embryo research, while the second approach is to establish new regulations for hSCBEMs based on their developmental potential. Both approaches are problematic. Firstly, the current regulations for embryo research result from historical and political compromises in most jurisdictions, while assessment of developmental potential is problematic due to the unavoidable uncertainty about the potential of any new hSCBEM and any changes converting it to a different model. These two papers emphasise the ethical, legal, and political minefield to be traversed in future research.

Comprehensive analytical technologies, including ‘omics’ and genetic tools, are now being widely applied to the field of implantation biology. **Shu-Yun Li and Francesco DeMayo (Contribution 6)** focus on the hormonal and gene regulation aspects of endometrial receptivity and implantation, and the recent progress provided by in vitro systems, comprehensive analytical (‘omics’) technologies, and genetic tools. They describe how advances in genetic manipulation, particularly the Cre/loxP system, have enabled the in vivo investigation of the role of genes in a uterine compartmental and cell type-specific manner, providing valuable insights into uterine biology during pregnancy and disease. The application of ‘omics’ technologies to analyse endometrial organoids has uncovered new molecular mechanisms and signalling pathways that regulate implantation. **Louie Ye and Eva Dimitriadis (Contribution 7)** provide a comprehensive overview of the wealth of data provided by modern ‘omics’ technologies, concerning endometrial receptivity and the window of implantation, including genomics, epigenomics, transcriptomics, proteomics, lipidomics, metabolomics, microbiomics, and integratomics. Their focus is to provide mechanistic insights into endometrial receptivity and their implications in infertility, highlighting advances and discussion of novel diagnostic and therapeutic strategies that may improve reproductive outcomes. It is concluded that various disciplines of the ‘omics’ have revolutionised our understanding of endometrial receptivity, uncovering key targets for advancing diagnostic and therapeutic strategies in reproductive health. For example, genomic studies linked SNPs in certain genes to poorer IVF outcomes, while different SNPs are implicated in recurrent implantation failure. Likewise, insights from other ‘omics’ identify potential targets for modulation. Clearly, there is an emerging foundation for developing personalised diagnostic and therapeutic strategies to enhance implantation success and improve reproductive outcomes. Integration of multi-‘omic’ datasets offers hope for a comprehensive understanding of the complex biological processes underpinning endometrial receptivity.

One family of conserved transcription factors, the HOX genes that have critical roles in reproductive tract development and endometrial functionality, is dealt with in detail in the paper by **Lorin-Manuel Pîrlog et al. (Contribution 8).** Their review highlights the molecular underpinnings of HOXA10/HOXA11 in reproductive health and their dysregulation in benign pathologies associated with infertility, such as endometriosis, adenomyosis, and endometrial polyps. The molecular mechanisms underlying their action include the modulation of extracellular matrix (ECM) remodelling via metalloproteinases, cytokines, and cell adhesion molecules. Aberrant HOX gene expression, driven by DNA hypermethylation or altered histone acetylation, compromises endometrial receptivity and implantation. HOXA10/HOXA11 genes have potential as biomarkers and therapeutic targets to optimise fertility outcomes and address reproductive pathologies.

No discussion of implantation is complete without a discussion of the embryo as a blastocyst. **Kathryn Gurner and David Gardner (Contribution 9)** discuss a critical aspect of implantation physiology, the role of metabolism, lactate, and pH regulation in signalling between blastocyst and endometrium. The blastocyst develops a unique metabolism that creates a specialised microenvironment at the site of implantation, characterised by high levels of lactate and reduced pH. Indeed, given its small size and high permeability, lactate may be the first embryonic signal received by the endometrium. While historically perceived as a metabolic waste product, lactate appears to serve as a signalling molecule that facilitates the invasion of surrounding tissues by cancers and promotes blood vessel formation during wound healing. The authors explore the origin and significance of blastocyst-derived lactate, its functional role at the implantation site, and how understanding this mediator of the maternal–foetal dialogue may help to improve implantation in assisted reproduction.

While this volume focuses largely on human reproduction, it is important to remember that there are many strategies for implantation utilised by other mammals. To varying degrees, implantation in all species includes alterations in epithelial polarity; the transformation of the endometrial stroma; the differentiation of the trophoblast; cell-to-cell and tissue-to-tissue signalling through hormones, cytokines, and extracellular vesicles; and the alteration of the maternal immune system. **Greg Johnson and colleagues (Contribution 10)** present a comparative view of implantation in livestock species in which there is considerable elongation of the conceptus during a long pre-implantation phase in the uterine cavity. This offers possibilities for studying molecular details of local interactions between trophoblast and uterine epithelium at implantation sites, which is notoriously difficult in species with small blastocysts like the human or the mouse. This review examines the unique features that ultimately lead to placental development in species that have epitheliochorial (pigs) and synepitheliochorial (sheep and cows) placentation, concluding with a listing of some important ‘omics’ studies that will underpin this new understanding.

In summary, throughout the series of papers, three themes are dominant: the development of 3D culture systems as the primary methods for resolving questions that cannot be studied in vivo; the progress from descriptive ‘omics’ to mechanistic insight; and implantation now being recognised as a complex, self-organising process and not merely the sum of individual molecular events.

The contributions bound together in this Special Issue of *Biomolecules* give an overview of the wealth of cell biological and molecular data on embryo implantation obtained in recent years. Advances in technologies and cultural techniques have enabled these findings. Biochemical regulatory processes are infinitely complex, involving multiple layers derived from the transcriptome, proteome, epigenome, microbiome, immunome and others, which can be both subtle and combinatorial. Functional studies using biological models with manipulations leading to gain or loss of function identify critical molecules but are restricted by the models used. Carefully proscribed clinical cohorts can provide essential information with artificial intelligence-assisted (AI) approaches now being applied. For example, when RNA seq was performed on endometrial biopsies from complex cohorts of women receiving IVF-ET, application of machine learning algorithms (AI) applied to the data enabled live birth prediction models, while drugs that may be beneficial in improving outcomes were developed [1]. This approach accounted for multiple confounders. Clearly, careful application of AI in the coming years will enable our field to progress through complex analyses in ways not previously possible.

A key criterion for whether cancer develops appears to be the organisational state of the tissue whose functions are propagated by a considerable level of self-organisation [2]. Given the complexity of the implantation site and its similarities to cancer development, benefit may be gained by taking a systems biology approach [3]: a step back from the detailed study of cellular and molecular events to look instead at the overall organisational state of the ‘implantation system‘ that includes the endometrium with its multiplicity of cell types, the trophoblast, and the microenvironment surrounding them, and how this develops to enable or not enable the establishment of a pregnancy. The multicellular organoids and co-culture systems now being developed offer opportunities for such an approach. The same logic that argues for using systems analysis approaches and organisational views must also apply to consider the ethical implications of the potential for self-organisation and the development of an organismic whole [4] in models of human implantation in vitro.

It is hoped that this volume may stimulate new generations to take up the challenge of exploring how successful implantation can be achieved.
